# Research of segmentation recognition of small disease spots on apple leaves based on hybrid loss function and CBAM

**DOI:** 10.3389/fpls.2023.1175027

**Published:** 2023-06-06

**Authors:** Xiaoqian Zhang, Dongming Li, Xuan Liu, Tao Sun, Xiujun Lin, Zhenhui Ren

**Affiliations:** College of Mechanical and Electrical Engineering, Hebei Agricultural University, Baoding, China

**Keywords:** hybrid loss function, CBAM, U-net, small spot segmentation, apple leaf disease

## Abstract

Identification technology of apple diseases is of great significance in improving production efficiency and quality. This paper has used apple Alternaria blotch and brown spot disease leaves as the research object and proposes a disease spot segmentation and disease identification method based on DFL-UNet+CBAM to address the problems of low recognition accuracy and poor performance of small spot segmentation in apple leaf disease recognition. The goal of this paper is to accurately prevent and control apple diseases, avoid fruit quality degradation and yield reduction, and reduce the resulting economic losses. DFL-UNet+CBAM model has employed a hybrid loss function of Dice Loss and Focal Loss as the loss function and added CBAM attention mechanism to both effective feature layers extracted by the backbone network and the results of the first upsampling, enhancing the model to rescale the inter-feature weighting relationships, enhance the channel features of leaf disease spots and suppressing the channel features of healthy parts of the leaf, and improving the network’s ability to extract disease features while also increasing model robustness. In general, after training, the average loss rate of the improved model decreases from 0.063 to 0.008 under the premise of ensuring the accuracy of image segmentation. The smaller the loss value is, the better the model is. In the lesion segmentation and disease identification test, MIoU was 91.07%, MPA was 95.58%, F1 Score was 95.16%, MIoU index increased by 1.96%, predicted disease area and actual disease area overlap increased, MPA increased by 1.06%, predicted category correctness increased, F1 Score increased by 1.14%, the number of correctly identified lesion pixels increased, and the segmentation result was more accurate. Specifically, compared to the original U-Net model, the segmentation of Alternaria blotch disease, the MIoU value increased by 4.41%, the MPA value increased by 4.13%, the Precision increased by 1.49%, the Recall increased by 4.13%, and the F1 Score increased by 2.81%; in the segmentation of brown spots, MIoU values increased by 1.18%, MPA values by 0.6%, Precision by 0.78%, Recall by 0.6%, and F1 Score by 0.69%. The spot diameter of the Alternaria blotch disease is 0.2-0.3cm in the early stage, 0.5-0.6cm in the middle and late stages, and the spot diameter of the brown spot disease is 0.3-3cm. Obviously, brown spot spots are larger than Alternaria blotch spots. The segmentation performance of smaller disease spots has increased more noticeably, according to the quantitative analysis results, proving that the model’s capacity to segment smaller disease spots has greatly improved. The findings demonstrate that for the detection of apple leaf diseases, the method suggested in this research has a greater recognition accuracy and better segmentation performance. The model in this paper can obtain more sophisticated semantic information in comparison to the traditional U-Net, further enhance the recognition accuracy and segmentation performance of apple leaf spots, and address the issues of low accuracy and low efficiency of conventional disease recognition methods as well as the challenging convergence of conventional deep convolutional networks.

## Introduction

Apples are rich in medicinal and nutritional value and are one of the most widely planted fruit industries in the world (Khan et al., 2022). From the data of recent years, the growth rate of apple production has been decreasing year by year (Liu et al., 2018), and analyzing the reasons for this, diseases are one of the important influencing factors. Diseases of apple trees occur in the roots, branches, fruits, and leaves, and most of them initially spread from the leaves, so accurate and effective identification of apple leaf disease types and the degree of disease is a key aspect of apple disease protection management. According to statistics, there are more than 100 kinds of apple leaf diseases, among which Alternaria blotch and brown spot disease are the two most common leaf diseases of apple trees. In this paper, we have segmented the spots and classified the diseases for the 2 common types of apple leaf diseases.

The traditional method of judging fruit tree leaf diseases mainly relies on expert experience by manually extracting the color, texture, and shape characteristics of diseased leaf images (Ayaz et al., 2021). However, in actual production activities, it is easy to misjudge the type of disease and thus misuse pesticides, which affects apple production. Therefore, a more convenient and accurate disease diagnosis method is urgently needed to analyze and determine the type of disease which provides researchers with a reasonable application strategy to prevent and control the disease on time and reduce the planting management pressure of fruit farmers. With the breakthrough progress of deep convolutional neural networks in classification tasks on open data sets, many scholars have applied image segmentation technology to the field of disease spot recognition to segment disease images and identify them in real-time, scientifically determine the type of leaf diseases and the degree of disease, take timely and effective measures to improve apple yield, and help fruit farmers achieve early disease control.

The current challenges of apple leaf and spot image segmentation can be summarized into the following three types:

1. Unbalanced pixel ratio. The disease spot information is readily lost in the disease spot segmentation task because the pixels in the diseased region only make up a small portion of all the pixels in the entire image. Additionally, because of the imbalanced pixel ratio, a lot of pixels in the background that can be classified easily hide a lot of the pixels in the rare diseased zone during the loss summing, which negatively affects model training and, as a result, the segmentation of diseased spots.
2. Hard example sample problem. The extraction of target leaf edges and disease areas is problematic in the natural environment due to leaf overlap, uneven lighting, and shadows. These difficult-to-classify pixels directly affect the outcomes of leaf segmentation, which in turn affects the extraction of disease spots.
3. When an apple tree is infected in its early stages, the fruit has not yet developed, and the illness first appears in the leaves. Brown to dark brown little round spots with a diameter of 2 to 3 mm was generated on the young leaves during the early stages of spotted defoliation, and purple haloes were frequently present surrounding the lesions with obvious margins. Yellowish-brown dots that eventually became circular emerged on the leaf surface in the early stages of brown spot disease. The early stages of spotted defoliation and brown spot are quite similar, making it challenging to tell them apart. This makes it difficult to identify the types of diseases, which has an impact on early disease prevention and control.

In order to more precisely locate disease areas and identify disease species, as well as to lay the groundwork for future assessments of the severity of disease in fruit trees and effective disease control methods, the main motivation for the current study is to segment the smaller spots on apple leaves and classify similar diseases. Smaller spots are challenging to identify in lesion segmentation, necessitating model improvement to enhance lesion segmentation performance. Early detection of apple leaf diseases is essential for timely disease management, illness prevention, and mitigation of effects on fruit quality and fruit yield. Further, the performance of various semantic segmentation models (such as Deeplabv3+, PSPNet (Pyramid Scene Parseing Network), and U-Net) in spot segmentation has been the focus of recent research, and performance evaluation measures like MIOU (Mean Intersection over Union), MPA (Mean Pixel Accuracy), Precision, and F1 scores were taken into account in the current work.

Too et al. (Too et al., 2019) compared various convolutional neural network models, including VGG16, InceptionV4, ResNet50, ResNet101, ResNet152, and DenseNets121, using plant leaf diseases from the publicly available Plant Village dataset as the research object. The results of the experiment revealed that the DenseNets network model performed the best in terms of classifying and identifying plant leaf diseases. Lin et al. (Lin et al., 2019) improved the UNet-based deep convolutional neural network model was proposed for cucumber powdery mildew to segment and extract the diseased areas of cucumber leaves with an average pixel accuracy of 96.08%, which is better than traditional detection methods such as K-means, random forest, and GBDT (Gradient Boosting Decision Tree). Zhong Y et al. (Zhong and Zhao, 2020) proposed three methods to identify apple leaf diseases: regression, multi-label classification, and Focal Loss function based on DenseNet-121 deep convolutional network, and the accuracy of the method on the test set was 93.51%, 93.31%, and 93.71%, respectively, which was better than the traditional CE (cross-entropy) loss function-based multi-classification methods. Santos et al. (Santos et al., 2020) used the Mask R-CNN instance segmentation network model to segment, detect and count the grape trees in the real scene, compared with other network models, the F-score of the Mask R-CNN network model achieved an optimal effect of 0.91. Ngugi et al. (Ngugi et al., 2020) modified the encoder component of the UNet network model to offer the network the ability of multi-scale feature extraction to achieve tomato leaf disease spot segmentation on complicated backdrops, thus increasing the segmentation accuracy of tomato leaf illnesses. On the entire plant leaf specimen dataset, Hussein et al. (Hussein et al., 2021) used DeepLabV3+ to conduct segmentation experiments and found that utilizing a deep learning semantic segmentation model produced superior semantic segmentation outcomes than target detection techniques like Faster R-CNN (Ren et al., 2017) and Yolo v5. Wang P et al. (Wang et al., 2021) proposed to use CA-ENet to identify different apple diseases. This method integrates a coordinate attention block in the EfficientNet-B4 network, uses deep separable convolution in the convolution module, and introduces the h-swish activation function. The experimental results show that the accuracy of this method is 98.92%, and the average F1 score is 0.988, which is better than ResNet-152, DenseNet-264, and ResNeXt-101. Tassis L M et al. (Tassis et al., 2021) used the Mask R-CNN network, U-Net, and PSPNet networks to automatically detect identify disease spots in field images containing some coffee trees and obtained 73.90% accuracy and 71.90% recall in the instance segmentation task; for U-Net and PSPNet networks, 94.25% and 93.54% average intersection and union were obtained. Li X et al. (Li et al., 2022) used U-Net, PSPNet, and DeepLabV3+ (Chen et al., 2018a) semantic segmentation model for potato leaf segmentation, and the MIoU of the model was 89.91% and MPA was 94.24%.

Studies have shown that plant leaf lesion segmentation based on deep learning semantic segmentation models is feasible, but existing studies have only used CNN-based models to identify crops and plant diseases without improving the models, and there are fewer studies on segmentation of apple leaf lesion regions based on semantic segmentation. Liu et al. (Liu et al., 2022) used the severity of apple Alternaria blotch assessed using DeeplabV3 +, PSPNet, and UNet. The correlation coefficient and consistency correlation coefficient were both 0.992 and the average accuracy of severity categorization was 96.41%. The study’s lack of many disease instances in a single leaf image was a drawback, even though the reference value and anticipated value were in agreement. In addition, in prior research, the loss function of the model is typically a single loss function. In this study, to enhance the segmentation performance and achieve more precise segmentation of leaves and disease spots under natural conditions, we fused two loss functions and added attention mechanisms to both the two effective feature layers extracted by the backbone network and the outcomes of the first upsampling.

Therefore, this paper has improved the U-Net model by adopting a hybrid loss function and adding an attention mechanism to perform pixel feature extraction and spot segmentation for two common types of apple leaf diseases, so that the disease can be recognized accurately. This method has improved the recognition accuracy and segmentation effect for small targets such as apple leaf spots while ensuring its feature extraction and classification ability.

The main contributions of this work are as follows:

1. Dice Loss and Focal Loss are combined as the loss function in this paper, causing the network to pay more attention to the similarity of lesions, increase the accuracy of image segmentation, and optimize the segmentation details.
2. The original U-Net model is proposed to be enhanced with an attention mechanism in this research. By comparing the segmentation accuracy after incorporating the three attention mechanisms SENet (Squeeze-and-Excitation Networks), ECANet (Efficient Channel Attention Module), and CBAM (Convolutional Block Attention Module), it is found that adding CBAM to the original model improves the network’s capacity to extract illness features and increases the robustness of the model.
3. The model in this work has the best segmentation performance in smaller disease spots segmentation recognition when the segmentation performances of Deeplabv3+, PSPNet, U-Net, and DFL-UNet+CBAM are compared.
4. The classification and identification of related diseases, as well as the segmentation and recognition of smaller disease spots in apple leaves, were accomplished. In general, the results of this experiment can serve as a technical foundation for the future segmentation, classification, and precise management of plant leaf disease spots.

The structure of the whole document is as follows. The first section of this essay provides an overview of the study context and topic’s importance, the research’s driving forces, its current state, its main contributions, and its primary ideas. In Section 2, the suggested modeling strategy is introduced, with an emphasis on the U-Net algorithm, the attention mechanism, and the loss function, as well as a description of the enhanced network topology. The study’s materials and procedures are described in Section 3, including the dataset preparation process, model training environment, model implementation platform, and an explanation of each model assessment metric’s parameter. The fourth section examines the experimental findings, investigates the segmentation impact of the model trained on smaller disease spots using a variety of algorithms, loss functions, and attention processes, and discusses the training strategy for the model with the best segmentation effect. The discussion of the research is introduced in Section 5, which mostly outlines the issues that need to be resolved. Section 6 summarizes the research of this paper and introduces the research conclusions of the test.

## Improved U-Net network structure

### U-Net network structure

One of the earliest full convolutional network-based image segmentation algorithms, U-Net is an upgraded semantic segmentation network built on FCN (Fully Convolutional Networks) (Shelhamer et al., 2017) and may maintain more local features in the segmentation outcomes.

The “U-shaped” symmetric encoder-decoder structure of the U-Net network’s second half, which is upsampling, is used for feature extraction in the first half. The enhanced feature extraction part of the process can be used to up-sample the five initial effective feature layers obtained from the backbone part and perform feature fusion to obtain an effective feature layer that fuses all features to classify each feature point. The backbone feature extraction part makes up the first half.

### Loss function

In this paper, a hybrid loss function was utilized to close the gap between the prediction results and the true values and achieved high confidence in the boundaries of segmented images. The commonly used loss function was CE Loss, but its role was relatively small when the examples were unbalanced. Lin, T.-Y. et al. (Lin et al., 2020) proposed focal loss to improve the accuracy of dense object detection. Dice Loss (Wang et al., 2020) and Focal Loss (Chen and Qin, 2022) were taken into consideration in order to address the issues of poor segmentation performance of smaller disease spots in apple leaves and the challenge of classifying apple Alternaria blotch and brown spot disease diseases with similar disease characteristics at the early stage of disease onset.

The basic idea behind Dice Loss was to measure the regional similarity between the prediction result and the true value during training; however, using Dice Loss directly reduced training stability. To avoid the problem of assigning different weights to the same class while ignoring the presence of hard examples in both positive and negative examples, such as pixels in the diseased area covered by raindrops and light or other leaf pixels in the background, the network was focused on learning hard examples by using a Focal Loss function that increases the loss value of challenging examples. By increasing the loss value of hard examples and forcing the network to concentrate on learning hard examples, it addressed the issue of unbalanced positive and negative examples as well as unbalanced hard and easy examples.

(1) CE loss is used to measure the difference between two probability distributions and the gap between model learning and reality. The traditional cross-entropy loss function is the most often used loss function in classification. Equation (1) displays its formula.


(1) $$CE\;Loss=-\left(y_{i}\log p_{i}+\left(1-y_{i}\right)\log\left(1-p_{i}\right)\right)$$

(2) Dice Loss places more emphasis on identifying leaf regions and gauges how well the outcomes anticipated and actual values in the area match up. Equation (2) illustrates the formula.


(2) $$Dice\;loss=\frac{2TP}{2TP+FN+FP}$$

where, correspondingly, TP (True Positive), FP (False Positive), and FN (False Negative) represent the total number of true positives, false positives, and false negatives.

(3) Focal Loss focuses the network on learning hard examples by enhancing the loss value of hard examples, balancing positive and negative examples and difficult and easy classification examples, as shown in equation (3).


(3) $$\text{Focal loss}\left(\text{Y},\text{P}\right)=-\frac{1}{\text{n}}\sum_{\text{i}=1}^{\text{n}}\left[{\text{αy}}_{\text{i}}{\left(1-{\text{p}}_{\text{i}}\right)}^{\text{γ}}\ln{\text{p}}_{\text{i}}+\left(1-\text{α}\right)\left(1-{\text{y}}_{\text{i}}\right){\text{p}}_{\text{i}}^{\gamma }\ln\left(1-{\text{p}}_{\text{i}}\right)\right]$$

In the equation, *n* stands for the total number of apple leaf samples, $y_{i}$ for the input sample’s true category, $p_{i}$ for the likelihood that the sample is 1, and γ for the modulation coefficient. The average logarithmic loss for each sample is shown by the logarithmic loss for all samples. To strengthen the focus on positive examples and improve the imbalance of targets in the case of extremely unbalanced categories, adding ${\left(1-p_{i}\right)}^{\gamma }$ will cause the loss value of samples with high prediction probability to decrease while the loss value of samples with low prediction probability to increase. Currently, image segmentation can only use it for binary classification. The positive example in the binary classification problem has a label of 1, and the negative example has a label of 0. For the positive example, the more $1-p_{i}$ , the harder it is to categorize the sample. The more $p_{i}$ is greater, the more challenging it is to classify negative examples.

In this study, the loss function employed a hybrid loss function (DFL) that scaled both Dice Loss and Focal Loss to the same order of magnitude to predict the input data, with Dice Loss emphasizing similarity and Focal Loss improving segmentation specifics to increase image segmentation accuracy.

### Attentional mechanism

Jain et al. (Jain et al., 2022) compared the Attention-UNet model with the UNet, UNet + + and UNet3P models, the AUC (Area Under Curve) value is 0.97, while the AUC values of other models are 0.964,0.966 and 0.965, respectively. The results show that the attention mechanism is beneficial to segment very bright and blurred plaque images that are difficult to diagnose using other methods.

The inclusion of an attention mechanism was thought to improve feature extraction because the leaf spot areas are smaller. The model would then assign different weights to each location of the input image and concentrate on the areas with crucial information, which would help it make more accurate judgments while using fewer resources. The attention mechanism has demonstrated strong performance in previous research on tasks like categorization, detection, and segmentation (Karthik et al., 2020; Mi et al., 2020). In this study, we thoroughly examined SENet (Hu et al., 2020), ECANet (Yu et al., 2022), and CBAM (Ma et al., 2022), three attention mechanisms, and we chose the best module to enhance apple leaf spot segmentation.

ECANet removed the two FC (Fully Connected) layers used in SENet and performed global average pooling without dimensionality reduction. It used the current channel and its k neighboring channels for local cross-channel interaction. SENet performed global average pooling of the input feature layer, took the Sigmoid after completing two full joins, obtained the weight of each channel of the input feature layer, and then multiplied that weight by the original input feature layer. Compared to SENet’s attention mechanism, which focused exclusively on channels, CBAM was a lightweight attention module that could be integrated into virtually any convolutional neural network, and almost negligible computation and parameters were introduced. It combined the channel attention mechanism and the spatial attention mechanism to jointly learn the important local detail information in the image, assign higher weights to the diseased spot region in the neural network’s feature map and lower weights to the background, improved the neural network’s attention to the diseased spot in the image, and then enhanced the network’s capacity for feature learning and expression.

In order to boost the network’s capacity to extract disease features and the resilience of the model, an attention mechanism was added to the two effective feature layers that the backbone network extracted, as well as to the outcomes of the initial upsampling.

### Improved U-Net network structure

This paper proposed an improved model based on U-Net that keeps the backbone feature extraction network but enhanced it by adding CBAM modules to the two effective feature layers extracted by the backbone network; after being subjected to feature fusion to complete two convolution operations, the effective feature layers obtained in the coding stage are then subjected to upsampling to recover the original image accuracy and detail information pixel by pixel. The CBAM attention module was then embedded after the first upsampling. The model was designed to recalibrate the weight relationships between features, amplify channel features of leaf disease spots, and suppressed channel features of healthy regions of leaves to improve the network’s ability to extract disease features and to increase the model’s robustness. The upper portion of the improved U-Net network was the backbone feature extraction network, and the lower portion was the enhanced feature extraction network.

**Figure 1:**
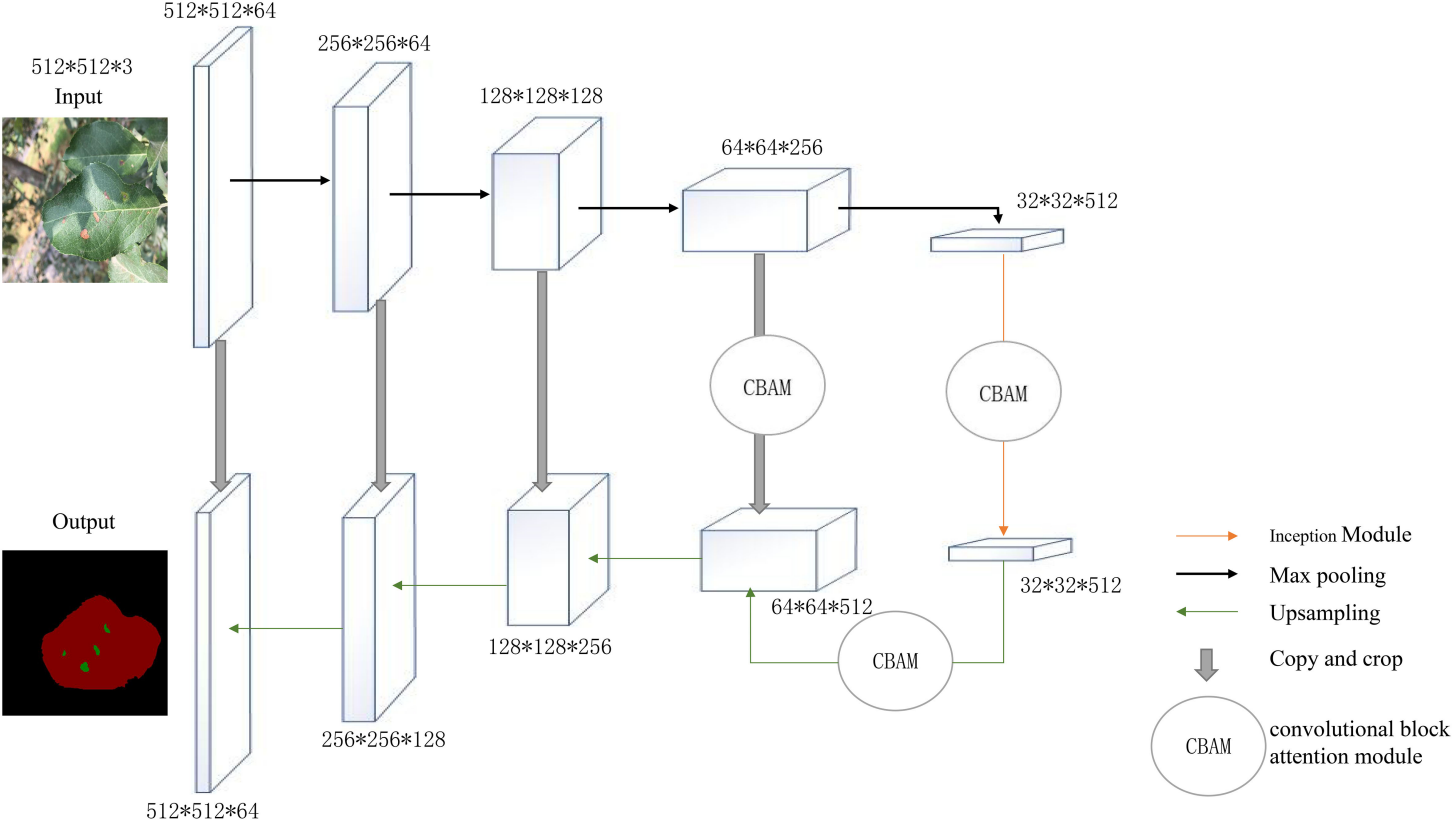
Improved U-Net network structure diagram.

Additionally, the improved model predicts the input data using a mixed loss function (DFL), which scales the focus loss and dice loss to the same magnitude. During training, Dice Loss focuses more on identifying the foreground region and assesses how closely the results of the prediction match the actual value in the area. By strengthening the loss value of hard examples (such as pixels in the diseased area covered by raindrops and light or other leaf pixels in the background), the Focal Loss function makes the network focus on the learning of difficult samples and solves the problem of imbalance between positive and negative examples and imbalance between difficult and easy samples. The structure of the network is shown in **Figure 1**.

## Materials and methods

### Dataset source

The image samples of apple leaf diseases in this experiment came from the public data set Plant Village (Geetharamani and Pandian, 2019). The dataset manually collects images of indoor and outdoor diseased apple leaves. In order to ensure the versatility of the model, outdoor landscape images were taken on sunny and rainy days, respectively.

**Figure 2:**
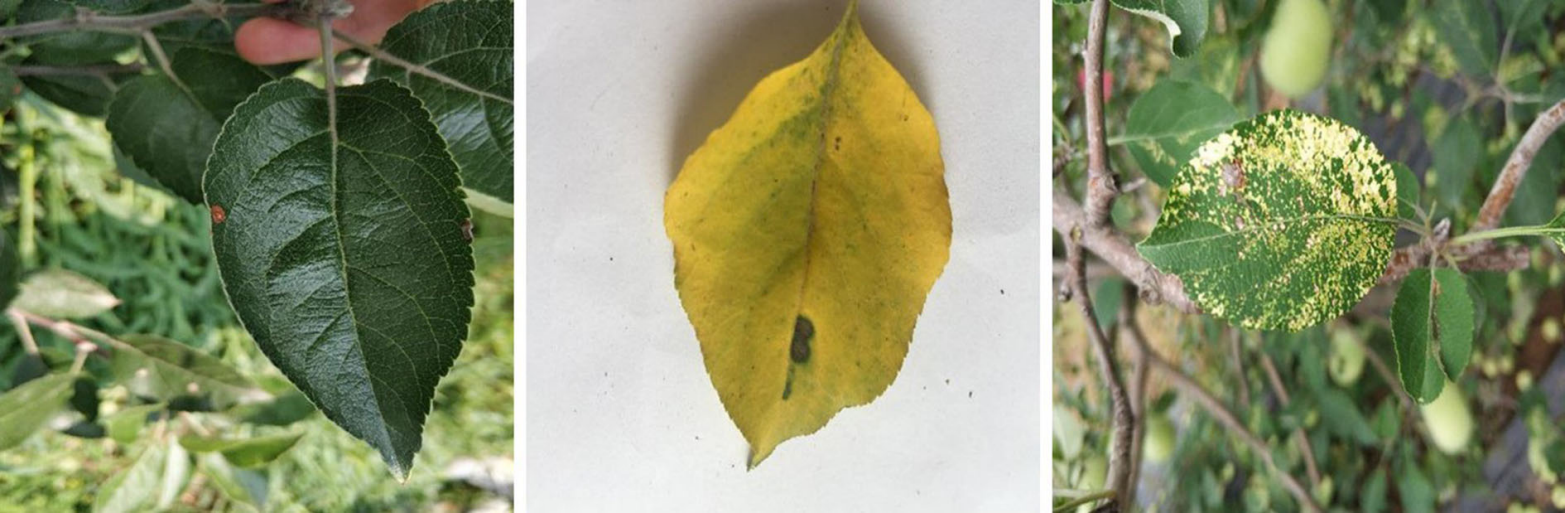
Examples of apple leaf disease image.

The data set in this paper contains different situations of a single leaf with a single disease and multiple diseases and multi-leaf images in complex backgrounds. As seen in **Figure 2**, the leaf diseases include single Alternaria blotch, brown spot, and multiple diseases (including brown spot and mosaic) of apple leaves. The samples in this dataset include pre-processing operations on the acquired images, such as image rotation, horizontal and vertical mirroring, a sharpness value, brightness value, contrast adjustment, and Gaussian blur on the original disease images. This pre-processing was done to prevent overfitting issues in the later network training phase, to improve the anti-interference ability of complex conditions as well as the generalization ability of the model, to increase the diversity, and to avoid generating problems during the network training phase, and thus the model robustness is enhanced.

Also to ensure a balanced sample, 1200 images of a single Alternaria blotch, 1200 images of a single brown spot, 600 images of apple leaf diseases infected with multiple diseases (mosaic and brown spot), a total of 3000 original images (JPG format) were selected, with a 1:1 ratio of complex background images to simple laboratory background images, which is more challenging than laboratory images of diseased leaves with simple backgrounds, with an original image size of 512 pixels * 512 pixels, and the dataset was divided into training, validation and test sets in a 6:2:2 ratio. As demonstrated in **Figure 2**, the image of apple leaf disease has the traits of a smaller disease spot and high similarity, which presents numerous difficulties for image segmentation.

For leaf segmentation, it is difficult to extract the target leaf’s edge because there are multiple leaves overlapping in the background of the image in the outdoor scene. Additionally, there are shadows in the leaf image due to uneven illumination, self-crimping, folding, and other factors, which makes the segmentation more challenging. Diverse and challenging to extract features from the target leaf scales. The extraction of disease spot features and the precise detection of disease spots are greatly hampered by the smaller disease spot pixels, which make up 0.2% to 0.4% of the leaf pixels in spot segmentation. Outside, there are materials that resemble spots that could prevent infections from being extracted. The segmentation impact of disease spots is easily influenced by the spots on raindrops and leaves.

### Dataset production

The photos must be converted into a dataset in PASCAL VOC format by the specifications of the model for the dataset. JPEGImages, ImageSets, and Annotations were the three main files that made up the PASCAL VOC format dataset.

The Segmentation folder of the ImageSets file contained four text files: train.txt, val.txt, test.txt, and trainval.txt, which, respectively, represented the training set, validation set, test set, and summary of the training and validation sets required by the model. The numbers of the photographs in each of the four text files’ respective sets, with each image number on a distinct line, made up their contents. To ensure the generalizability of the model, the image numbers were created at random.

**Figure 3:**
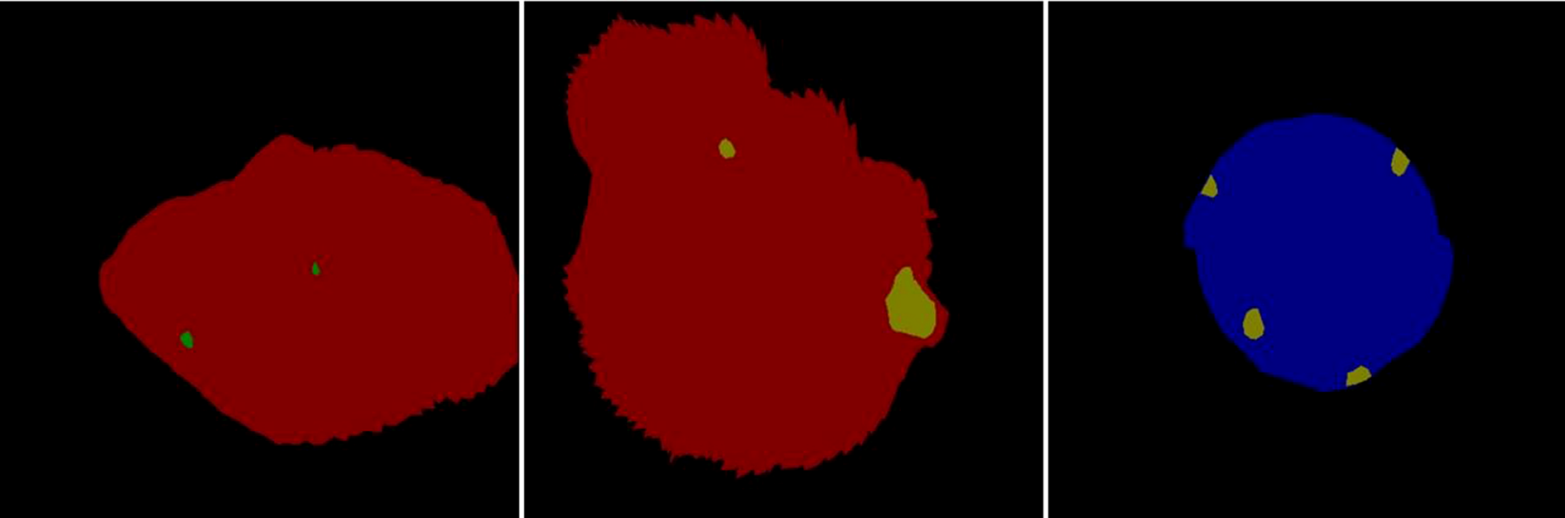
Examples image of a.png tag image.

The function of Annocations file was mainly to store the annotation information corresponding to the leaf image. In order to train the model, a large number of data annotations of the data set must be performed; this work used Labelme as the data labeling software. The annotation file is initially stored in.json format, and then changed to a tag image in.png format by batch converting the file, as shown in **Figure 3**.

### Setting up the testing environment and parameters

Intel Core i7-9700, 32 GB of RAM, and an Nvidia GeForce RTX 2080Ti graphics card were the specifications of the computer’s processor. Model construction, training, and prediction were performed in this deep learning environment using Tensorflow-gpu1.13.2, keras2.1.5, Windows 10, 64-bit operating system, Python 3.6.13 compiled environment, CUDA10.1 architecture, and cuDNN7.4.1 Development library. When compared to other adaptive learning rate algorithms, the Adam approach is simple to use, very computationally efficient, memory-light has a quicker convergence time and is invariant to diagonal gradient rescaling. In order to select the model with the best segmentation effect through interaction, this experiment trains the models using the Adam optimizer until convergence.

During training, the input image is 512*512 pixels. Padding=1 is utilized so that each input square can serve as the convolution window’s center and stride=1 is used to limit the number of input parameters and processing. The output size is the same as the input when stride and padding are both set to 1. In order to nonlinearly transform the input, the activation function needs to be introduced. The activation function used in this paper is sigmoid. The whole training is divided into two stages, the freezing stage and the thawing stage. The quantity of images entered into the network at once during training is referred to as the batch size. The model training generation is known as an epoch. It can be regarded as a suitable training generation when there is a minimal difference in error between the training set and the test set. In order to ensure that the model achieves the best effect in terms of accuracy and training time, this paper sets the training generation to 200. According to the graphics performance of the operating system and the size of the image, the first 50 stages are the freezing stage, the batch size is set to 4, and the last 150 stages are the thawing stage, the batch size is set to 2, to ensure that the model achieves the best effect in terms of accuracy and training time, and avoids insufficient memory. The average value of the updated network weight in the algorithm is the initial learning rate. The maximum learning rate is set at 0.0001 in order to speed up the model training’s transition into a stable learning state. The learning rate is reduced by the cosine annealing attenuation method. Period = 5 is set during training to attenuate the model once every 5 epochs and preserve it, preventing the loss of the training model in the event of a power outage or an abnormal exit during long-term training.

### Model evaluation indicators

This study evaluated the classification accuracy of the model for the disease classification problem using true positive (TP, the number of times the model accurately predicts the disease type), true negative (TN, the number of times the model accurately predicts the leaf area), false positive (FP, the possibility of misjudging the leaf area as the spot area), and false negative (FN, the possibility of misjudging the spot area as the leaf area).

After the establishment of the model, it was necessary to evaluate its effectiveness. This work suggested using the mean intersection ratio MIoU (Shoaib et al., 2022), the category average pixel accuracy MPA, the precision rate Precision, and the comprehensive evaluation index F1 Score (Shoaib et al., 2022) as the evaluation index of the segmentation results in order to quantify and assess the model’s performance.

To facilitate the interpretation of the evaluation metric formulas, it is assumed that the data set has a total of k + 1 categories. $p_{ij}$ denotes the number of pixels for which category i is predicted to category j, $p_{ii}$ denotes the number of pixels that are correctly predicted, and $p_{ij}$ and $p_{ji}$ denote the number of false negative and false positive pixels, respectively.

(1) MIoU

The average of the ratio between the intersection and concatenation of the set of pixels whose true value is the spot and the set of pixels whose predicted value is the spot is determined, as indicated in equation (4). The higher the MIoU value, the higher the overlapping degree between the projected spot area and the actual spot area.


(4) $$\text{MIoU}=\frac{1}{k+1}\sum_{i=0}^{k}\frac{p_{ii}}{\sum_{j=0}^{k}p_{ij}+\sum_{j=0}^{k}p_{ji}-p_{ii}}$$

(2) MPA

Equation (5) demonstrates that MPA is the average of the percentage of total pixels that fall into the proper prediction category.


(5) $$\text{MPA}=\frac{1}{k+1}\sum_{i=0}^{k}\frac{P_{ii}}{\sum_{j=0}^{k}P_{ij}}$$

(3) Precision

The accuracy rate is defined as the proportion of actual diseased pixels to those predicted as such by the model, as indicated in equation (6). Less false detection areas are seen in the prediction results as the value increases.


(6) $$\text{Precision}=\frac{\text{TP}}{\text{TP+FP}}$$

(4) Recall

The recall rate, also known as the check-all rate, is the proportion of spots that are detected to all spots in the data set, as is evident from equation (7).


(7) $$\text{Recall}=\frac{\text{TP}}{\text{TP+FN}}$$

(5) F1 Score

The F1 Score metric combines the Precision and Recall outputs, as given in equation (8). F1 Score accepts values between 0 and 1. The model’s best output is represented by 1, while its worst output is represented by 0. The more correctly recognized spot pixels, the more accurate the segmentation result.


(8) $$F1=\frac{2*\text{Precision}*\text{Recall}}{\text{Precision+Recall}}$$

## Test results and analysis

### Test process

**Figure 4:**
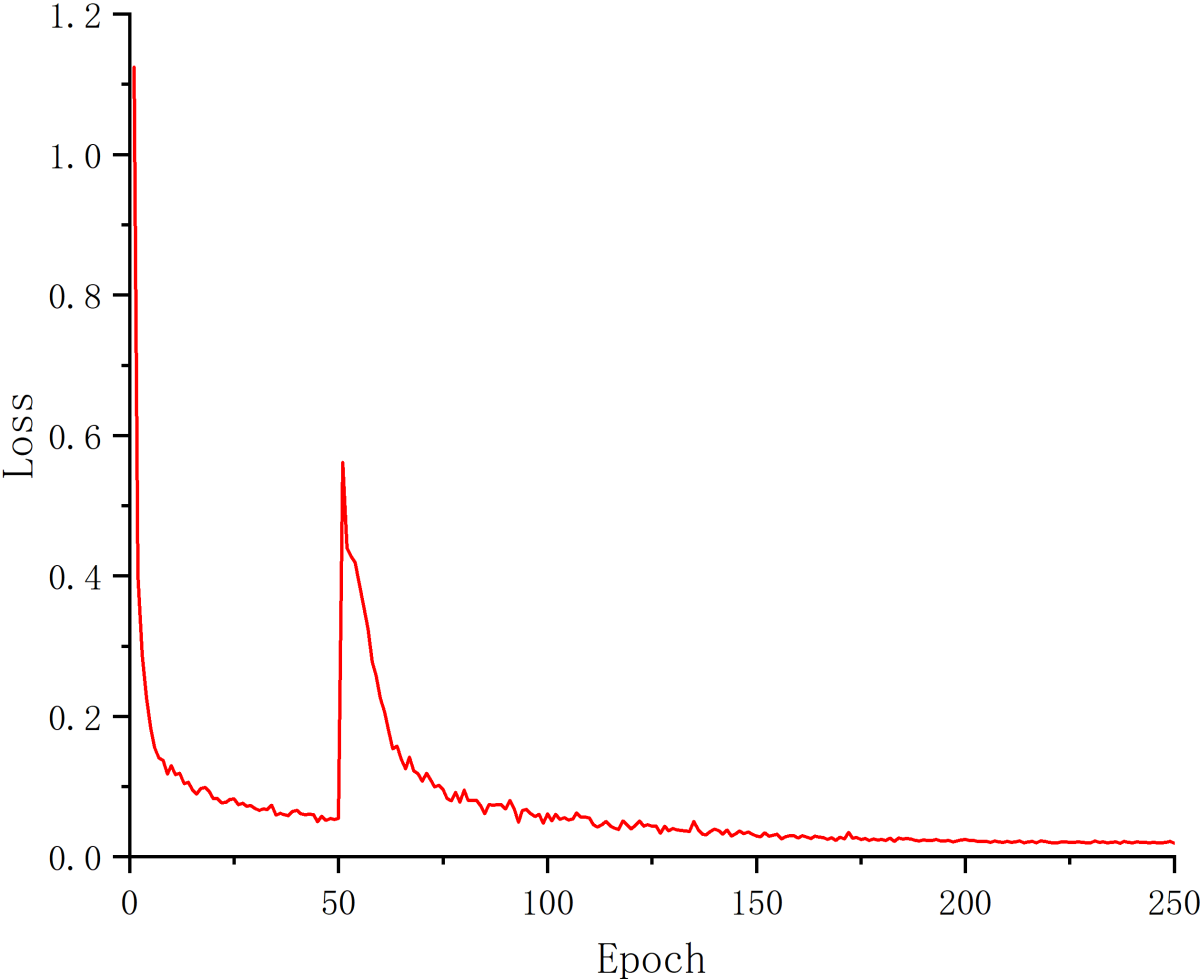
Loss curve.

After 250 epochs of training the U-Net model, the Loss finally converged to 0.022. **Figure 4** depicts the Loss’s evolution throughout training epochs. It is clear from the figure that the Loss stopped dropping and stabilized around 200 epochs, indicating that the model had progressively converged at that point. The U-Net model with 200 and 250 epochs of training was chosen to compare the test results in order to determine the best model for this experiment. The findings are displayed in **Table 1**.

**Table 1:** Comparison of segmentation results for different epochs of training.

Epoch	MIoU/(%)	MPA/(%)	Precision/(%)
200	89.11	94.52	93.53
250	88.96	94.30	93.24

**Table 1** shows that as training epochs increased, MIoU, MPA, and Precision values fell at training 250 epochs, indicating the occurrence of an overfitting phenomenon. As a result, the model in this research was chosen for training 200 epochs.

In the experiment to segment unhealthy spots, the target pixel points can be separated into two primary categories: diseased spots and healthy parts. Since the background does not include any diseased spots, it is likewise segmented into healthy parts. Three loss functions—CEL, CEDL, and DFL—are employed in this study’s ablation experiments, with U-Net serving as the main body. The experiment assesses the effectiveness of the loss functions using the loss rate and accuracy of the validation set. **Table 2** presents the outcomes.

**Table 2:** Experimental results of loss function ablation.

Loss function	Network	Val-acc/(%)	Val-loss	MIoU/(%)	MPA/(%)
CEL	Original U-Net	98.34	0.063	89.11	94.52
CEDL	Original U-Net	98.37	0.055	89.33	94.85
DFL	Original U-Net	98.40	0.010	90.09	95.14
CEL	Improved U-Net	98.51	0.039	90.89	95.07
CEDL	Improved U-Net	98.76	0.045	89.96	94.90
DFL	Improved U-Net	98.86	0.008	91.07	95.58

**Figure 5:**
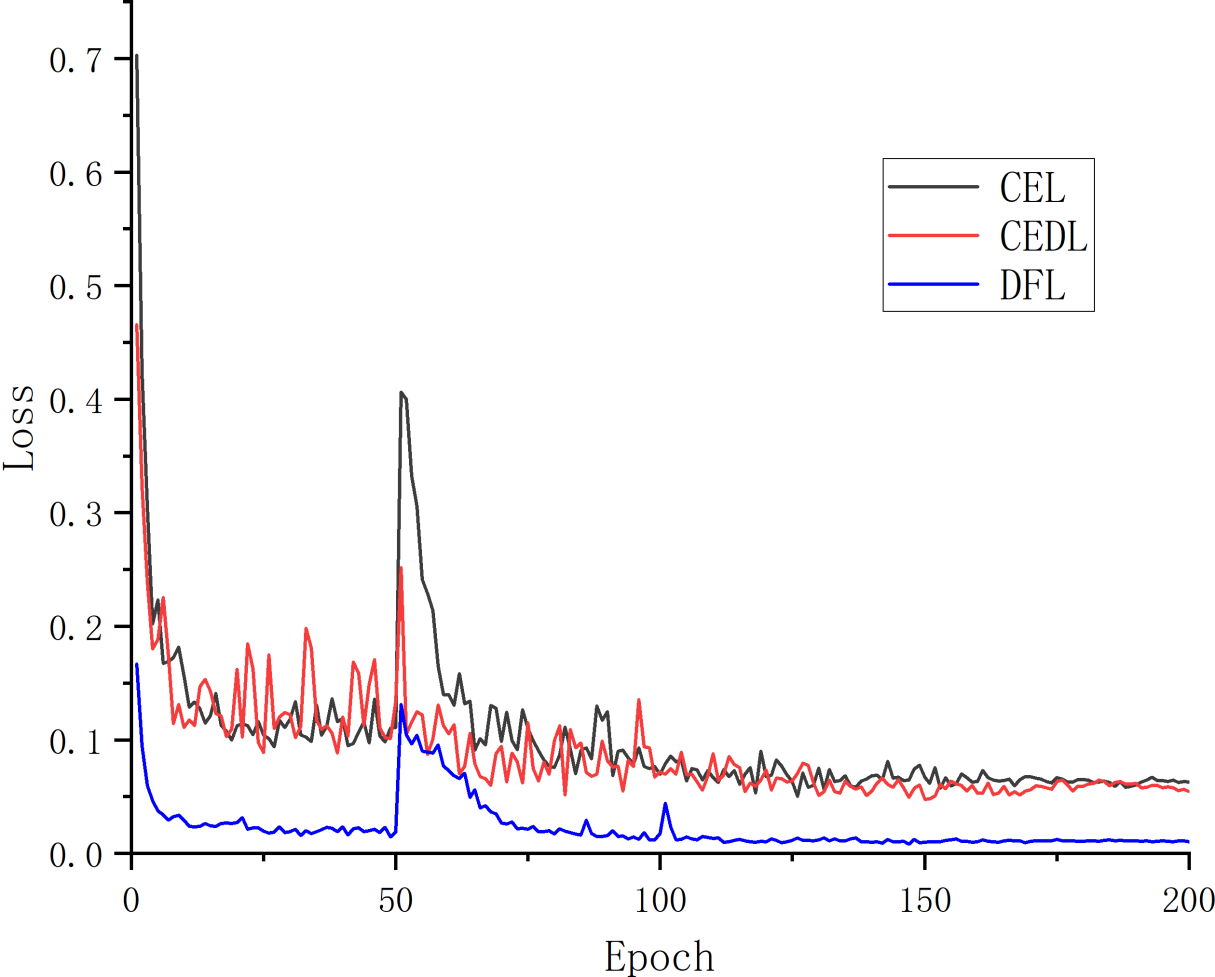
Loss curves of different loss functions.

**Table 2** compares the performance of the original U-Net and the improved U-Net deep learning designs using various loss functions. Verification loss, accuracy, MIoU, and MPA are employed as evaluation indicators for these variables. As can be observed, under the presumption that picture segmentation accuracy is guaranteed, the outcomes of the four parameters are 0.008, 98.86%, 91.07%, and 95.58%, respectively, after adding the DFL mixed loss function. The modified model’s average loss rate dropped from 0.063 to 0.008; the lower the loss, the more accurate the model, the MPA increased by 1.06%, the prediction category correctness increased, the MIoU score rose by 1.96%, and the more the predicted illness area overlapped with the actual disease region. The challenge of distinguishing apple Alternaria blotch disease and brown spot disease with high similarity in the early stage of disease is resolved by the addition of the DFL mixed loss function, which also addresses the issue of poor segmentation performance of smaller disease points. Additionally, it lessens the disparity between simple and difficult training examples as well as the disparity between positive and negative training examples. The process by which the effective loss value of the U-Net model changes when different loss functions are applied is shown in **Figure 5**. The outcomes demonstrate that the DFL mixed loss function employed in this study has the smallest loss value, the fastest decline rate, and the smoothest training procedure.

The model is trained by adding various attention mechanism modules using the same experimental setting and training parameters as U-Net combined with hybrid loss function DFL. The experimental findings are displayed in **Table 3** to compare the various types of segmentation MIoU.

**Table 3:** Comparison of the cross-merge ratio (MIoU) for each category of the model after adding the attention mechanism.

Segmentation Model	leaf/(%)	Alternaria blotch/(%)	Brown spot disease/(%)
DFL-UNet	93.70	76.05	84.12
DFL-UNet+SENet	94.05	78.56	84.93
DFL-UNet+ECANet	93.43	78.99	85.11
DFL-UNet+CBAM	94.86	79.02	85.28

As shown in **Table 3**, the accuracy of disease identification can be increased by adding SENet or ECANet, but the addition of the CBAM attention mechanism results in superior disease identification. The comparison shows that the MIoU value of smaller Alternaria blotch disease spots increases by 2.97%, indicating that the CBAM attention mechanism can effectively focus on the disease spots in the image and suppress the interference information. To address the issue of the DFL-UNet model’s poor segmentation performance of smaller spots, we decided to integrate the CBAM module in this study.

### Results analysis

The segmentation performance of the Deeplabv3+ model, PSPNet model, original U-Net model, and DFL-UNet+CBAM model was compared, and the results are shown in **Table 4**. In this paper, MIoU, MPA, and F1 Score were all used as evaluation metrics for segmentation results under the same research object and the same experimental conditions.

**Table 4:** Comparison table of segmentation performance of different models.

Model	MIoU/(%)	MPA/(%)	F1 Score/(%)
Deeplabv3+	85.94	92.04	91.80
PSPNet	83.49	86.81	90.40
U-Net	89.11	94.52	94.02
DFL-UNet+CBAM	91.07	95.58	95.16

**Figure 6:**
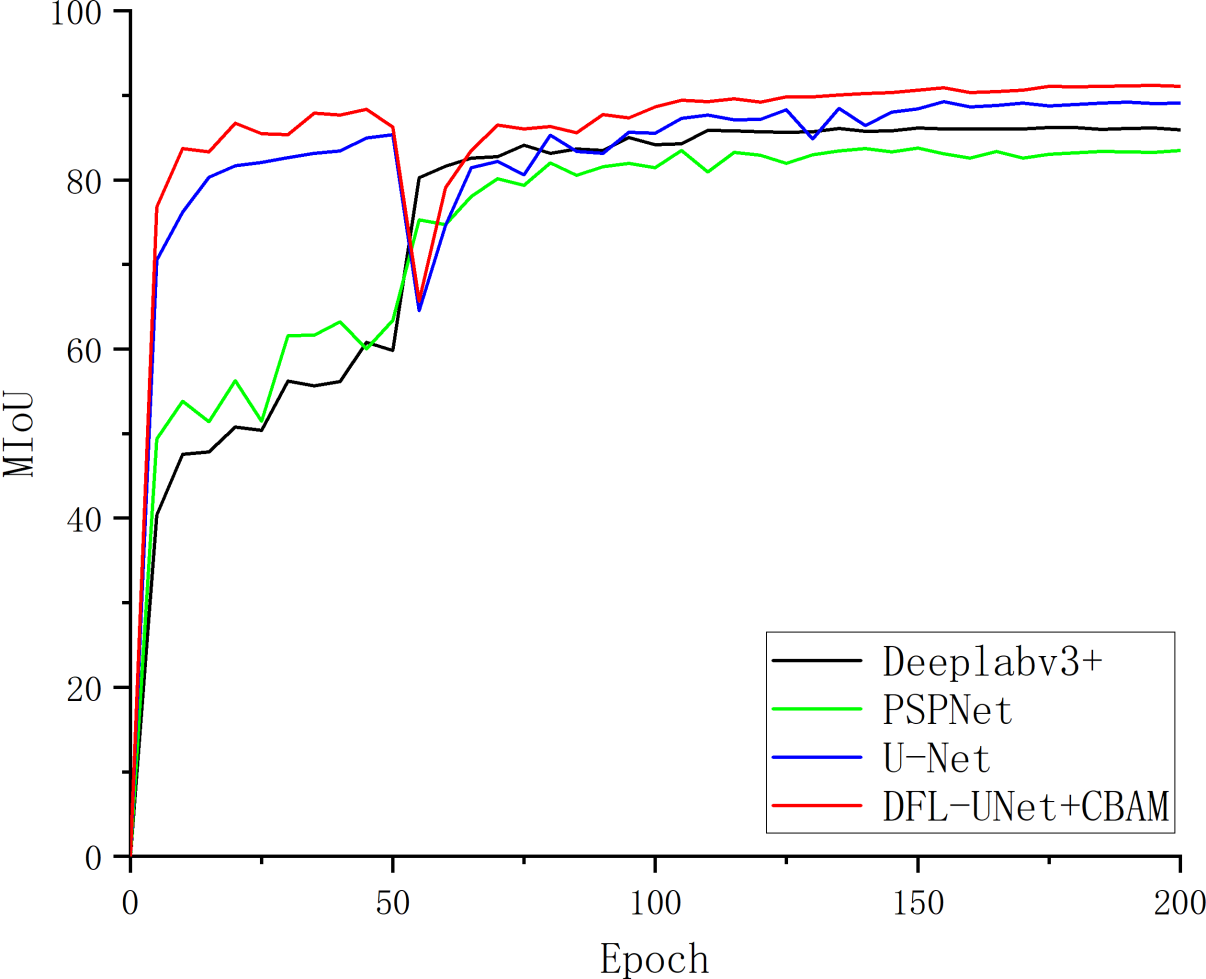
Comparison of different model segmentation mean intersection over union.

As can be seen in **Table 4**, when comparing the four models, the MIoU, MPA, and F1 Score of the DFL-UNet+CBAM model proposed in this paper are the highest, increasing by 1.96% in MIoU value, 1.06% in MPA value, and 1.14% in F1 Score when compared with the original U-Net model. This shows that the model in this paper correctly identifies the most diseased pixels and can effectively optimize the segmentation results and obtain more. The change in MIoU value during model training is depicted in **Figure 6**, and it is also obvious from the change curve that the model used in this paper has the greatest MIoU value, suggesting the highest overlap between the predicted spot area and the actual spot area.

**Table 5** compares the segmentation performance of smaller spots before and after model modification using MIOU, MPA, Precision, Recall, and F1 scores as assessment metrics. This comparison is done to indicate the benefit of the suggested method in recognizing smaller spots.

**Table 5:** Analysis of quantitative results of U-Net and improved U-Net.

Model	Disease types	MIoU/(%)	MPA/(%)	Precision/(%)	Recall/(%)	F1 Score/(%)
Original U-Net	Alternaria blotch	74.61	84.93	86.02	84.93	85.47
	Brown spot	84.1	92.7	90.06	92.7	91.36
Improved U-Net	Alternaria blotch	79.02	89.06	87.51	89.06	88.28
	Brown spot	85.28	93.3	90.84	93.3	92.05

As can be seen from **Table 5**, compared to the original U-Net model, the segmentation of Alternaria blotch disease, the MIoU value increased by 4.41%, the MPA value increased by 4.13%, the Precision increased by 1.49%, the Recall increased by 4.13%, and the F1 Score increased by 2.81%; in the segmentation of brown spots, MIoU values increased by 1.18%, MPA values by 0.6%, Precision by 0.78%, Recall by 0.6%, and F1 Score by 0.69%. The spot diameter of the Alternaria blotch disease is 0.2-0.3cm in the early stage, 0.5-0.6cm in the middle and late stages, and the spot diameter of the brown spot disease is 0.3-3cm. Obviously, brown spot spots are larger than Alternaria blotch spots. The segmentation performance of smaller disease spots has increased more noticeably, according to the quantitative analysis results, proving that the model’s capacity to segment smaller disease spots has greatly improved.

**Figure 7:**
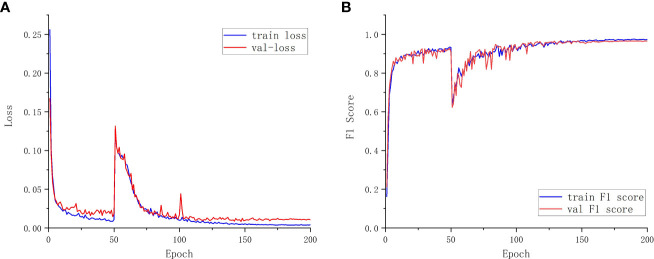
Training and validation set details. **(A)** Loss function curve. **(B)** F1 score curve.

Additionally, the proposed model’s training and validation performance are assessed using the training set F1 score, validation set F1 score, a training set loss, and validation set loss. This is done to further validate the performance of the model segmentation. The loss value is used to quantify the discrepancy between the model’s true value and its predicted value, and the F1 score is calculated as a weighted average of Precision and Recall metrics. Better model robustness is associated with smaller loss functions. The training score determines the generalization ability of the algorithm in its training samples. The verification score determines the optimal model (Srinivasu et al., 2022). **Figure 7** displays the model’s performance in relation to the hyperparameters.

In this study, we used a trained semantic segmentation model to predict apple leaf disease in laboratory and field environments. The image dataset must meet two criteria: first, it must allow for the simultaneous occurrence of various illnesses on the same leaf; and second, it must allow for the presence of complicated backgrounds in some images to guarantee the data images’ excellent generalization ability.

**Figure 8:**
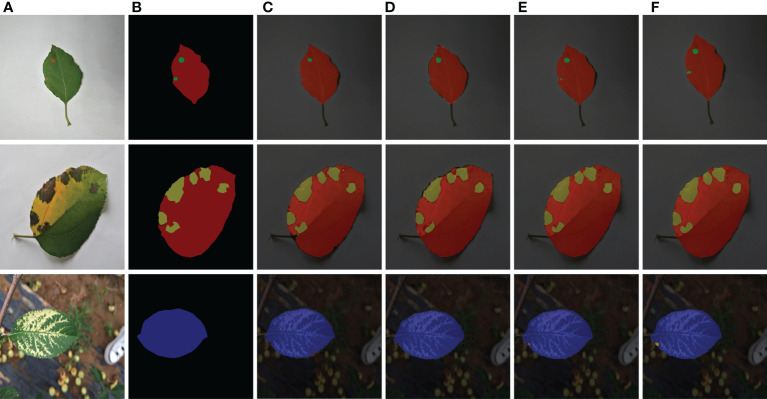
Comparison of segmentation results of various models. **(A)** Original images. **(B)** Ground truth. **(C)** Deeplabv3+ segmentation results. **(D)** PSPNet segmentation results. **(E)** U-Net segmentation results. **(F)** Improved U-Net segmentation results.

In comparison to the Deeplabv3+ model, the PSPNet model, and the original U-Net model, the segmentation results of the DFL-UNet+CBAM model utilized in this paper are shown in **Figure 8** for the test set of apple disease leaf photos.

The prediction outcomes of single-leaf spot segmentation against various backgrounds are shown in **Figure 8**. **Figure 8** shows it abundantly clear that the network structure suggested in this paper achieves more accurate segmentation for apple leaf spots and produces better segmentation results for both the disease location on the leaf and the size of the spot area. This network structure is also more accurate than other networks used in this paper. When recognizing brown spot disease, the Deeplabv3+ model in **Figure 8C** incorrectly recognized the green halo area surrounding the illness spot as the disease spot; the PSPNet model in **Figure 8D** has a condition where the object boundary segmentation is discontinuous and the segmentation result is rough, the border between the leaf and the backdrop is hazy, and there is a missing section for the area affected by the brown spot disease. Analysis of the segmentation results of the model proposed in this paper demonstrates that the model in this paper can segment the semantic objects completely, finely, and accurately, and it is apparent from **Figure 8F** that the recognition results of the disease spots and the segmentation results of the edges of the disease spots in this paper are more accurate.

Comparing **Figure 8C** and **Figure 8D**, it can be seen that the network structure of the proposed model performs well in the segmentation of smaller spots. Although the U-Net model in **Figure 8E** identified smaller spots in the apple Alternaria leaf spot and brown spot categories of foliar diseases, the identified spot area was incomplete. In contrast, the model in **Figure 8F** accurately identified the smaller spots in the categories of apple ringspot and brown spot, and the recognition results were more accurate.

## Discussion

Semantic segmentation and attention mechanisms have been widely used in the realm of disease recognition. An ASPP (Atrous Spatial Pyramid Pooling)-based DeepLabV3+ semantic segmentation network model, for instance, was developed by Li L et al. (Li et al., 2023). The experimental findings revealed that the model’s average pixel accuracy (MPA) and average intersection (MIoU) reached 97.26% and 83.85%, respectively. Additionally, Li Q et al. (Li et al., 2021) proposed an integrated U-Net segmentation model for small sample datasets, merging U-edge Net’s features and high-level features using ASPP. The experimental findings demonstrated that the method significantly increased the segmentation accuracy of the target fruits as well as the model’s capacity for generalization.

The segmentation task of apple leaves and spot areas was carried out in this study using three traditional semantic segmentation network models (DeepLabV3+, PSPNet, and U-Net). The segmentation performance of the model was evaluated throughout the experiment. Also, the performance of the model is addressed in relation to the implications of various loss functions and attention mechanisms. Following are our findings:

1. Three semantic segmentation network models (DeepLabV3+, PSPNet, and PSPNet) were compared and their segmentation and convergence capabilities for the apple leaf and speckle regions were examined. The findings indicate that PSPNet and Deeplabv3+ are not as effective in segmenting data as the U-Net network model.
2. Investigated is how the U-Net network model chooses its loss function. According to the results, the addition of the DFL hybrid loss function improves the segmentation performance and classification capacity of the model. The average loss rate val-loss lowers from 0.063 to 0.008, the MIoU index increases by 1.96%, and the MPA increases by 1.06%.
3. Compare the different U-Net attention mechanism modules. The findings demonstrate that the addition of the CBAM attention mechanism improves the disease recognition effect. Comparatively, it is discovered that the MIoU value of the smaller speckle leaf spot disease spot is increased by 2.97%, demonstrating that the CBAM attention mechanism can concentrate on and pay attention to the disease spot in the image, as well as effectively suppress the interference information, which enhances the model’s focus on the target channel and spatial information.

In the prior research, the loss function of the model is typically a single loss function. In this study, to enhance the segmentation performance and achieve more precise segmentation of leaves and disease spots under natural conditions, we fused two loss functions and added attention mechanisms to both the two effective feature layers extracted by the backbone network and the outcomes of the first upsampling.

**Figure 9:**
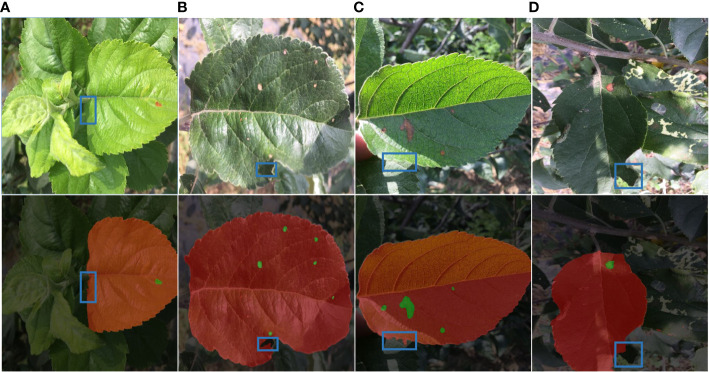
Detection fault analysis. **(A)** Leaf occlusion. **(B)** Self-crimp factor. **(C)** Leaf folding. **(D)** Insufficient light.

Overall, our technique demonstrates good adaptability in the single background and complicated background segmentation and detection of leaf spots. But because there are so many distractions in the natural world (such as uneven lighting), incorrect detection and missed detection will always happen there. In order to test the segmentation performance of the model, **Figure 9** uses the relatively smaller and more challenging-to-identify Alternaria blotch disease as an example. It then displays the segmentation prediction results of the diseased leaves and disease spots in the multi-leaf image in the natural environment. The findings demonstrate that the disease spot segmentation effect is effective when the uneven light shadow coverage is varied, however, there is a false detection part between the leaf and the background.

The target leaves’ edges are difficult to extract because the background of the image in the outdoor scene has multiple leaves overlapping each other. Additionally, there are shadows in the leaf images due to uneven lighting or because of curling and folding, which makes the segmentation more challenging.

The area of light irradiation to the leaves is also diverse, as illustrated in **Figure 9**, due to different shooting angles and self-curling factors. As a result, diseased leaves are concealed by other leaves or object shadows, which causes the pigment imbalance problem. Diagram 9 (a) (b) The disease leaf identification is insufficient because only a small portion of the disease leaf’s edge was impacted by other leaves, a phenomenon known as missed detection; in **Figure 9**(c), the disease leaf edge segmentation is inaccurate because there is cross-over between leaves and a light uneven dual impact; as seen in **Figure 9**(d), the diseased leaf shadow is heavier Part of the incorrect check for the background area, the overall image tone is dark, the color of the measured target is distorted.

The following issues still need to be resolved even though we explored the segmentation recognition of smaller spots in apple leaves in this work and increased the segmentation effect and recognition accuracy of smaller spots.

1. The ability to quickly diagnose diseases in fruit trees is crucial for practical production, so future research should focus on enhancing the network structure to reduce the model segmentation time. This will help fruit farmers quickly confirm the diagnosis of diseases in fruit trees and quickly apply pesticides.
2. In actual, there are frequently several leaves in a single image and the leaves are set against a complicated background. The presence of disease spots on many leaves is not taken into account in this work. Consequently, to enhance the segmentation performance of disease leaves and thereby enhance the precision of disease spot recognition, the model needs to be further enhanced in the upcoming research.
3. The actual development of disease species is complex and varied. Despite the fact that the method described in this paper enhances the segmentation performance of smaller spots in apple leaf diseases and the recognition precision of difficult-to-classify diseases, the disease species in the training data set still need to be increased, and the disease species can be increased later to improve the recognition and segmentation ability of the model for various diseases and make the model broadly applicable.

## Conclusion

In practice, the naked eye can easily misinterpret the type of disease and thus overuse pesticides, which in turn affects apple production. Therefore, disease diagnosis must be easier, faster, and more accurate, while the type of disease must be analyzed and determined. Apple leaf spot is very small and has similar characteristics when the disease first appears, while the actual orchard environment has different light conditions, overlapping leaf shade, etc. A deep learning-based apple leaf disease spot segmentation technique is suggested for apple leaf disease recognition by utilizing CNN’s strong feature extraction capabilities in order to minimize the influence on disease spot segmentation. The core network architecture used by the method is a convolutional neural network called U-Net, and to better extract picture features, its structure and parameters have been modified and optimized. The identification of apple leaf disease depends directly on the precision of the segmentation method. In order to address the issues of low recognition accuracy and subpar performance of smaller spot segmentation in apple leaf disease recognition, this paper uses apple leaf Alternaria blotch and brown spot as its research object. It then proposes a method of spot segmentation and disease recognition based on hybrid loss function and CBAM. The following conclusions were obtained from the study:

1. To deal with the issue of poor performance in segmenting smaller spots in apple leaves, a model for apple leaf disease segmentation based on hybrid loss function and CBAM network has been developed. Firstly, the model using mixed loss function of Dice Loss and Focal Loss has swapped out the original cross entropy function, which has given larger weight to the samples that are difficult to classify, making the model pay more attention to the target with smaller pixel proportion. Secondly, the backbone network’s two useful feature layers and the outcomes of the first upsampling have been combined with the CBAM module to complete the extraction of pixel features and disease spot segmentation for apple Alternaria blotch and brown spot. This has caused the model to pay more attention to the regions with important information.
2. MIoU values in DFL-UNet+CBAM model employed in this study were 91.07%, MPA values o were 95.58%, and F1 Score values were 95.16%. These values were higher than those of the original U-Net model by 1.96%, 1.06%, and 1.14% respectively, and the illness identification impact was also enhanced. The segmentation result images have also shown that the DFL-UNet+CBAM model has had better segmentation and recognition capabilities, can more precisely identify smaller disease spot areas, improves the detection and recognition accuracy of smaller disease spots, better satisfies the requirements of apple leaf disease recognition, and provides a basis for the diagnosis of apple leaf diseases.
3. In the multi-blade environment of nature, several leaves may coexist on a single map, and various illnesses may be present on the leaves. The experimental results demonstrate that the semantic segmentation model of apple leaf diseases trained in this paper using a single leaf dataset can not only detect a single background in the laboratory but can also be used to detect apple leaf diseases in the complex background of the natural environment; it can not only detect single objects of single and multiple leaves, but it can also detect multiple objects of single leaves, demonstrating powerful segmentation performance.

Research demonstrates that the model can ensure segmentation accuracy in complicated orchard environments as well as laboratores, particularly when it comes to the edge segmentation accuracy of smaller disease spots. The suggested method performs segmentation better than other methods, and the model has good generalizability. In the future, it might serve as a technical foundation for the segmentation, categorization, and precise management of plant leaf disease spots.

## Data availability statement

The original contributions presented in the study are included in the article/supplementary material. Further inquiries can be directed to the corresponding author.

## Author contributions

XZ drafted the original draft. XZ and DL reviewed and revised the original draft. XLiu provided guidance. TS and XLin collected the data. ZR provided financial supports and retouched the manuscript. All authors contributed to the article and approved the submitted version.
